# Acknowledgement to the reviewers of GMS Journal for Medical Education

**DOI:** 10.3205/zma001583

**Published:** 2023-02-15

**Authors:** Martin R. Fischer, Götz Fabry

**Affiliations:** 1 GMS Journal for Medical Education, Chief Editor, Erlangen, Germany; 2 University Hospital, LMU Munich, Institute for Medical Education, Munich, Germany; 3 Albert-Ludwig-Universität Freiburg, Institut für Medizinische Psychologie und Soziologie, Freiburg, Germany

## Acknowledgement

We gratefully acknowledge the valuable contribution of the following peer reviewers, who supported our Journal in 2022. We are looking forward to continuing our collaboration!

## Reviewers in alphabetical order

- Christian Abshagen, Windisch
- Kambiz Afshar, Hannover
- Matthias Angstwurm, München
- Tsedeke Asaminew, Jimma (ET)
- Cadja Bachmann, Rostock
- Ivan Bank, Amsterdam (NL)
- Daniel Bauer, Bern (CH)
- Nicola Bauer, Köln
- Jan Becker, Münster
- Marianne Behrends, Hannover
- Sven Benson, Essen
- Pascal Berberat, München
- Tanja Birrenbach, Bern (CH)
- Kerstin Bitter, Berlin
- Mozhgan Bizhang, Witten
- Sebastian Bode, Ulm
- Christoph Bohne, Frankfurt/Main
- Hans Martin Bosse, Düsseldorf
- Cindy Brandes, Osnabrück
- Barbara Braun, Mannnheim
- Andreas Breuer-Kaiser, Bochum
- Christoph Broding, Witten
- Monika Brodmann Maeder, Bern (CH)
- Irene Brunk, Berlin
- Petra Brzank, Nordhausen
- Heinz Hans Florian Buchner, Wien (A)
- Rainer Büscher, Essen
- Jean-Francois Chenot, Greifswald
- Iris Demmer, Göttingen
- Jana Deppermann, Oldenburg
- Andreas Dinkel, München
- Carsten Döing, Düsseldorf
- Fabian Dupont, Homburg
- Jan P. Ehlers, Witten
- Maren Ehrhardt, Hamburg
- Tobias Eichinger, Zürich (CH)
- Yannick G. Eller, Bern (CH)
- Caroline Elliott, Coventry (UK)
- Gundula Ernst, Hannover
- Rebecca Sarah Erschens, Tübingen
- Mona Eulitz, Witten
- Folkert Fehr, Sinsheim an der Elsenz
- Teresa Festl-Wietek, Tübingen
- Sabine Ingrid Fischbeck, Mainz
- Volkhard Fischer, Hannover
- Michael Freitag, Oldenburg
- Martin Gartmeier, München
- Robert Gaschler, Hagen
- Nadja Gebhardt, Heidelberg
- Waltraud Georg, Augsburg
- Heide Götze, Leipzig
- Joachim Graf, Tübingen
- David Groneberg, Frankfurt/Main
- Claudia Gundacker, Wien (A)
- David Gurrea Salas, Windisch
- Jennifer Guse, Hamburg
- Stefan Gysin, Luzern (CH)
- Martin Haag, Heilbronn
- Petra Hahn, Freiburg
- Eckhart G. Hahn, Erlangen
- Houda Hallal, Köln
- Jonathan Harth, Witten
- Wolf Hautz, Bern (CH)
- Stephan Heermann, Freiburg
- Inga Hege, Augsburg
- Patrick Heger, Ulm
- Heike Heiß, Ulm
- Linn Hempel, Halle/Saale
- Eva Hennel, Bern (CH)
- Martina Heßbrügge, Essen
- Monika Himmelbauer, Wien (A)
- Margarethe Hochleitner, Innsbruck (A)
- Anke Hollinderbäumer, Mainz
- Christopher Holzmann-Littig, München
- Angelika Homberg, Mannheim
- Mekonnen Asfaw Hussen, Addis Abada (ET)
- Sören Huwendiek, Bern (CH)
- Nana Jedlicska, München
- Laura Jung, Berlin
- Robert Jungwirth, Ulm
- Martina Kadmon, Augsburg
- Yassin Karay, Köln
- Christin Kleinsorgen, Hannover
- Fee Klupp, Heidelberg
- Katharina Knie, Freiburg
- Timm Knörr, Heidelberg
- Thomas Kollewe, Frankfurt/Main
- Felix Krause, Aachen
- Dana Kropff, Würzburg
- Susanne Kühl, Ulm
- Raphael Kunisch, Erlangen
- Katrin Kunze, Osnabrück
- Ute Lange, Bochum
- Wolf Langewitz, Basel (CH)
- Christoph Lanzen, Mainz
- Ronny Lehmann, Heidelberg
- Dorothea Lemke, Frankfurt/Main
- Nicola Litke, Heidelberg
- Henriette Löffler-Stastka, Wien (A)
- Sabine Ludwig, Berlin
- Karoline Lukaschek, München
- Cornelia Mahler, Tübingen
- Jan Matthes, Köln
- Juliane Mees, Würzburg
- Susanne Michl, Berlin
- Valeria Milani, Fürstenfeldbruck
- Equlinet Misganaw, München
- Andreas Möltner, Heidelberg
- Tobias Mühling, Würzburg
- Laura Müller, Ulm
- Beate Müller, Köln
- Brigitte Müller-Hilke, Rostock
- Oliver Münsterer, München
- Ede Nagy, Heidelberg
- Joachim Neumann, Halle/Saale
- Dino Carl Novak, Berlin
- Wolfgang Öchsner, Ulm
- Rüdiger Ostermann, Münster
- Saskia Pante, Heidelberg
- Dorothea Penders, Berlin
- Swetlana Philipp, Jena
- Maximilian Philipp, Frankfurt/Main
- Alexander Rahman, Hannover
- Patrick Rebacz, Witten
- Alexander Renkl, Freiburg
- Fabian Riedel, Heidelberg
- Caroline Riedel, Heidelberg
- Christina Rieger, Germering
- Bernd Romeike, Rostock
- Marco Roos, Augsburg
- Fredy-Michel Roten, Bern (CH)
- Miriam Rothdiener, Tübingen
- Thomas Rotthoff, Augsburg
- Martin Ruff, Lütjenburg
- Emma C. Ryan, New Haven (USA)
- Christian Scheffer, Witten
- Kristina Schick, München
- Ann-Kathrin Schindler, Augsburg
- Falk Schlegelmilch, Göttingen
- Achim Schneider, Ulm
- Michael Schön, Ulm
- Eva Schönefeld, Münster
- Christian Schulz, Berlin
- Johannes Schulze, Frankfurt/Main
- Katrin Schüttpelz-Brauns, Mannheim
- Simon Schwill, Heidelberg
- Roland Seifert, Hannover
- Thomas Shiozawa-Bayer, Tübingen
- Elizabeth Sierocinski, Greifswald
- Anne Simmenroth, Würzburg
- Melanie Simon, Aachen
- Robert Speidel, Ulm
- Thomas Stadtmüller, München
- Robin Stark, Saarbrücken
- Verena Steiner-Hofbauer, Wien (A)
- Beate Stock-Schröer, Witten
- Patrick Strasser, Ulm
- Fabian Stroben, Berlin
- Cynthia Szalai, Mülheim a.d. Ruhr
- Daniel Tolks, Hamburg
- Gert Ulrich, Zürich (CH)
- Christian Vajda, Graz (A)
- Sonya E. Van Nuland, New Orleans (USA)
- Wolfgang von Gahlen-Hoops, Kiel
- Yvonne Wagner, Stuttgart
- Michaela Wagner-Menghin, Wien (A)
- Robin Walter, Bern (CH)
- Stefan Watzke, Halle/Saale
- Marc Weidenbusch, München
- Jürgen Westermann, Lübeck
- Marie-Christin Willemer, Dresden
- Anja Zimmermann, Berlin
- Jan Zottmann, München
- Michaela Zupanic, Witten

